# Organic additives used in beef cattle feedlot: Effects on metabolic parameters and animal performance

**DOI:** 10.1111/asj.13183

**Published:** 2019-03-22

**Authors:** Rhaony Gonçalves Leite, Eliéder Prates Romanzini, Lutti Maneck Delevatti, Alvair Hoffmann, Adriana Cristina Ferrari, André Pastori D'Aurea, Lauriston Bertelli Fernandes, Amanda Prates Oliveira, Ricardo Andrade Reis

**Affiliations:** ^1^ São Paulo State University (Unesp) School of Agricultural and Veterinarian Sciences Câmpus Jaboticabal Department of Animal Science Jaboticabal SP Brazil; ^2^ Premix^®^Company Ribeirão Preto SP Brazil

**Keywords:** average daily gain, digestibility, growth promotants

## Abstract

Organic additives are recently being used in animal diets owing to their ability to control metabolic issues and result in better animal performance. Specifically, the organic additive Fator P^®^ presents an additional advantage that is to cause a lesser greenhouse gas emission. This study evaluated whether Fator P^®^ intake changes ruminal parameters or animal performance of beef cattle. Evaluations were carried out in a feedlot experiment divided into growing (46 days; two diets [control mix—CM and standard mix—SM] and finishing (lasted 83 days; four diets: CM, SM, Fator P^®^ + virginiamycin, and Fator P^®^ alone [FP]) trials. Animal performance study involved 48 animals allocated to 12 collective pens in completely randomized experimental design. Ruminal parameters were evaluated in separate metabolism study developed carried out using individual pen with four steers. During growing trial, FP diet resulted in higher (*p* < 0.05) dry matter intake (DMI) and ruminating time. In the finishing trial, diets containing Fator P^®^ resulted in higher DMI than obtained with CM. Most of the ruminal parameters did not differ (*p* > 0.05) among dietary treatments. Therefore, Fator P^®^ represents a viable and safe strategy for supplementation to beef cattle finished using high‐concentrate diet in feedlot systems.

## 1 Introduction

Brazil is one of the largest beef cattle producers in the world. Beef cattle are produced in extensive systems in which the use of technology and human interference are minimal, resulting in very low productivity indexes (Lobato et al., 2014). However, the adoption of feedlot technology by beef cattle farmers has led to improvements in the beef quality and increases in the productivity of the system. In Brazil, beef cattle production is divided clearly into two separate phases (Souza, Pereira, Ribeiro, Santos, & Valadares Filho, 2014). The first occurs from birth until weaning, while the second occurs from weaning until slaughter. However, the transition between the first phase (in pastures) and the second phase (normally developed in feedlots, where diets contain considerable amounts of energy) can result in metabolic disturbances that reduce the animals’ performance and, consequently, economic outcomes.

This transition, termed the adaptation phase in feedlots, can affect production and, consequently, the productive results. The use of feed additives would help directly in controlling the metabolic parameters to ensure optimal animal conditions during this phase (Hernández, Benedito, Abuelo, & Castillo, 2014; Jouany & Morgavi, 2007). These additives should control the microbial population and thereby prevent abrupt changes in the ruminal parameters, which can cause ruminal acidosis and reduce animal performance (Anderson, Schneider, Erickson, MacDonald, & Fernando, 2016). However, the traditional additives used are compounds that are prohibited in some countries; this prohibition can include the final products (e.g., milk, meat) from animals fed the additive‐supplemented diet. Therefore, organic additives are recently being used owing to their benefit of being composed of organic ingredients that are permitted in all countries. These organic additives can also control metabolic parameters and result in better animal performance during the adaptation phase (Patra & Saxena, 2009). Specifically, Fator P^®^ has been proven to have the previously cited benefits as well as the additional advantage of causing less greenhouse gas emission (close to 17%), as reported by Fernandes, D'Aurea, and Fernandes (2015).

We hypothesized that the use of an organic additive (Fator P^®^) in a feedlot diet would not affect the dry matter intake (DMI), nutrient digestibility, ruminal parameters, or animal performance during the adaptation and total phases. Nellore cattle (young bulls and steers) were used to test this hypothesis.

## 2 Materials and methods

The experiment was divided into two studies: one for animal performance, and the other for animal metabolism. The animal performance study was conducted in the Forage Crops and Grasslands section of São Paulo State University, “Júlio de Mesquita Filho” (Unesp) (Jaboticabal, São Paulo, Brazil). The protocol used was approved by the Ethics, Bioethics, and Animal Welfare Committee of Unesp, Jaboticabal (Protocol number 12703/15). The animal metabolism study was performed by Premix^®^ Company (Patrocinio Paulista, São Paulo, Brazil).

### 2.1 Experimental diets and chemical analyses

The animal performance study was divided into the growing trial (46 days) and the finishing trial (83 days). The experimental diets used in this study (Table 1) were formulated according to National Research Council recommendations (NRC, 2000) for the finishing trial of bulls, with total digestible nutrients of 69.9% and 74.1% for the growing and finishing trials, respectively. For the animal metabolism study, four experimental diets were used during the finishing trial. In all studies, the diets were supplied twice a day (07:00 and 14:00 hr) for the entire experimental period, with ad libitum feed intake by the animals.

**Table 1:** Chemical composition of the experimental diets used for the different feedlot trials and the chemical composition of diets

Variable	Experimental diet	
	Growing trial^a^	Finishing trial^b^
Ingredients (% DM)		
Sugarcane bagasse	17.86	12.95
Corn grain (ground)	69.15	78.27
Soybean meal	8.99	5.03
Mix	4.00	3.76
Chemical composition (% DM)		
Dry matter	84.41	85.83
Organic matter	78.34	78.57
Crude protein	14.88	11.89
Neutral detergent fiber	48.80	39.58
Acid detergent fiber	16.67	12.83
INDF	12.21	10.62

a Growing trial: 46 days from the start.

b Finishing trial: 83 days from the final growing trial until slaughter.

DM, dry matter; INDF, indigestible neutral detergent fiber.

During the growing trial, two concentrates were used: a control mix (CM) of 0.244 g/kg dry matter (DM) monensin + 0.195 g/kg DM virginiamycin, and a standard mix (SM) of 5.848 g/kg DM Fator P^®^ + 0.122 g/kg DM monensin. Fator P^®^ is composed of amino acids (lysine, methionine, and tyrosine at 16.40, 2.98, and 3.00 g/kg, respectively), the vitamin choline (40 g/kg), minerals (chrome and zinc organics at 0.05 and 1.05 g/kg, respectively), the probiotic *Saccharomyces cerevisiae* (7 × 10^8^ CFU/kg), and essential fatty acids (linoleic and oleic acids at 108.9 and 99 g/kg_,_ respectively).

The roughage:concentrate ratio used in the finishing trial was 18:82 in all diets, and was stepped up during the initial 12 days with corn silage, sugarcane bagasse (roughage), and concentrate until the final ratio was obtained. The diets were changed after 2 days during the step‐up period, where the corn silage:sugarcane bagasse:concentrate ratios were 32:18:50, 27:18:55, 22:18:60, 17:18:65, 12:18:70, 7:18:75, and 0:18:82 (final ratio), respectively. The different diets contained similar quantities of nitrogen (14.6% crude protein [CP]) and total digestible nutrients (69.9%).

**Figure 1:**
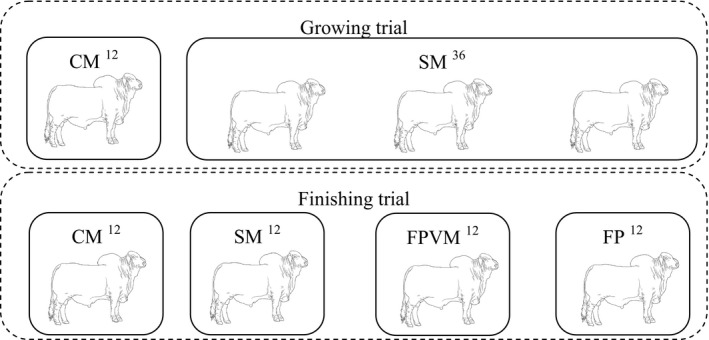
Treatments for each group of bulls (four bulls per collective pen) inside the collective pen (total = 12) during the growing and finishing trials. CM = control mix of 0.244 g/kg DM monensin + 0.195 g/kg DM virginiamycin; SM = standard mix of 5.848 g/kg DM Fator P^®^ + 0.122 g/kg DM monensin; FPVM = 5.848 g/kg DM Fator P^®^ + 0.195 g/kg DM virginiamycin; and FP = 5.848 g/kg DM Fator P^®^. Twelve and 36 are the number of animals allocated to each treatment

The finishing trial occurred after the growing trial and included four diets with a roughage:concentrate ratio of 13:87. The diet treatments were CM, SM, 5.848 g/kg DM Fator P^®^ + 0.195 g/kg DM virginiamycin (FPVM), and 5.848 g/kg DM Fator P^®^ alone (FP). The animals fed SM during the growing trial (36 animals) were divided into three groups of 12 animals (SM, FPVM, and FP—Fig. 1) each for the finishing trial, whereas the CM group remained unchanged. These diets contained similar quantities of nitrogen (13.4% CP) and total digestible nutrient (74.1%).

The samples of diets and feed refusals obtained from the digestibility trial were oven dried at 55°C for 72 h and then ground using a Wiley mill with a 1‐mm sieve. Thereafter, the samples were stored before the analyses to determine the DM (method ID 934.01) and mineral matter (method ID 942.05) as described by the Association of Official Analytical Chemists (AOAC, 2006). The nitrogen concentration was determined using a LECO FP‐528 nitrogen analyzer (LECO Corp., St. Joseph, MI).

The neutral detergent fiber (NDF) content was determined using alpha‐amylase, without the addition of sodium sulfite, as described by Van Soest, Robertson, and Lewis (1991), applying the Ankom 200 Fiber Analyzer (Ankom Technology, Fairport, NY). The acid detergent fiber content was determined according to the method described by Goering and Van Soest (1970), using the Ankom 200 Fiber Analyzer. The indigestible NDF concentrations of the diet samples were determined with the method described by Casali et al. (2008). Samples were weighed (0.5 g) into F57 filter bags, placed within the rumen of a cannulated steer for 240 h, and subsequently analyzed for NDF as described above.

### 2.2 Animals and measures of animal performance

Nellore young bulls (*N* = 48), weighing 394.1 ± 6.3 kg, with an average age of 24 months, were used. The feedlot contained 12 collective pens, in each of which four animals were allocated (Fig. 1). Each treatment used three collective pens and 12 animals, where each animal was used as a repetition for calculations. The collective pens had a semi‐roof and communal water bowls and feed bunks.

All variables were measured in each trial and during the total period of 129 days. To evaluate the individual DMI in kilograms per day (kg/day), the feed was weighed daily (being the refusals from the excess diet provided considered into calculation) in each collective pen and then divided by the total number of animals in the pen. To assess the animal performance, the animals were weighed monthly, always in the morning before the diets were supplied. The aim was to measure the average daily weight gain (ADG). Specifically, at the start and end of the feedlot period, the animals were fasted for 12 h to obtain the final ADG. After determining the individual DMIs, the feed conversion ratio (FCR) was calculated.

To evaluate the apparent digestibility (AD), fecal samples were collected from each animal at different time points (07:00; 12:00, and 17:00 hr) during three consecutive days. Dietary samples were also collected during the same period. The daily fecal and dietary samples were stored at −18°C. At the end of the digestibility trial, a composite sample was created for each animal. To determine the daily intake and AD of the DM and nutrients, the feces were analyzed to determine the DM, CP, and NDF concentrations as described by Goering and Van Soest (1970), Van Soest et al. (1991), and AOAC (Association of Official Analytical Chemistry) (2006). The following equation was used to measure the nutrient apparent digestibility: AD = [(nutrient intake—excreted nutrient)/nutrient intake] × 100, with the nutrient intake being measured from the dietary samples (nutrient in diet supplied—nutrient in refusals).

### 2.3 Feeding behavior

To measure feeding behavior during the growing trial, the animals were observed at every 10 min for 11 h (07:00–18:00 hr). The evaluation was performed during this period to determine whether some treatments led to increased rejection of the feed bunk compared with other treatments. The behavioral categories evaluated were the eating time, ruminating time (RT), nonchewing behavior (NB), and drinking time (DT).

According to Robles, Ganzález, Ferret, Manteca, and Calsamiglia (2014), the eating time is defined as when the animal is eating with its muzzle in the feed bunk or chewing or swallowing food with its head over the bunk. The ruminating time involves the regurgitation, mastication, and swallowing of the bolus. Nonchewing behaviors occur when the animal is resting, and no chewing behavior or apparent activity is being performed. The drinking time is the duration of which the animal has its muzzle in the water bowl or is swallowing the water.

Data for each activity are presented as minutes per day, with the day being considered as the daily period with solar light. The results represent the average for each activity performed by animals inside each collective pen, multiplied by the total time of observation, measured in minutes (660 min), divided by the total observations during the entire period (67 observations).

### 2.4 Animals and measurements of animal metabolism

Four Nellore steers with the rumen cannulated were used for this study. These animals had an average weight of 600 kg and age of 3 years. The study was divided into four periods of 21 days each. The animals were adapted to the experimental diets for 16 days (finishing trial diets), where the last 5 days were used for sample collection and measurements. The animals were fed in collective pens containing a collective feed bunk and water bowl.

The pH and ammonia nitrogen (NH_3_‐N) and volatile fatty acid (VFA) contents in the rumen were measured at 24 h after feeding. Sampling was performed before feeding (0 h), and at 2, 4, 8, 16, and 24 h after feeding. During the sampling days in each experimental period, the ruminal fluid was harvested from three different sections of the rumen to represent this ecosystem. The ruminal fluid was filtrated, and its pH was measured using a pH meter (DM‐23‐DC model; DIGIMED, Digicrom Analytic, São Paulo, Brazil). The filtered sample was stored (−18°C) for further analyses.

The rumen NH_3_‐N content was measured via the calorimetric method as described by Chaney and Marbach (1962). To evaluate the VFAs in the rumen, the stored samples were thawed in a refrigerator overnight and then centrifuged at 4°C and 20,000 *g* for 30 min. The supernatant was analyzed for VFAs using the method described by Palmquist and Conrad (1971), with a GC2014 gas chromatography system (Shimadzu Corporation, Kyoto, Japan) equipped with an HP‐INNO wax capillary column (30 m × 0.32 mm; Agilent Technologies, Loveland, CO) and operated at an initial temperature of 80°C and a final temperature of 240°C.

### 2.5 Statistical analysis

A completely randomized experimental design was used for the animal performance study, with two treatments for the growing trial and four treatments for the finishing trial (1 and 3 degrees of freedom, respectively). When significant, the means of treatments were compared using Tukey's test with 5% significance. The General Linear Model procedure of SAS 9.1 (SAS Institute, Cary, NC) was used. Importantly, because intrinsic animal effects can result in residual confounding in the analysis of variance, the total feedlot period of the animals was considered in the evaluations.

For the animal metabolism study, a Latin square (4 × 4) experimental design was used, with four treatments and four periods. The variables were analyzed as repeated measurements using the MIXED procedure of SAS 9.1. The treatments and times were considered as fixed effects in the Latin square, and the animals were considered as randomized effects. Differences among means were determined using Tukey's test, with significance defined at 5%.

## 3 Results

Differences (*p* < 0.05) in the eating time and ruminating time were observed during the growing trial (Table 2). Animals fed with CM spent more time eating (208.51 min) than did those fed with SM (157.89 min). Conversely, the ruminating time was greater for animals fed with SM (76.07 min) than for those fed with CM (47.61 min). With regard to the other activities, there were no differences (*p* > 0.05) in the nonchewing behaviors and drinking times among the different treatments.

**Table 2:** Feeding behaviors of animals during the first days of the growing trial

Activity (minutes)	Treatment^1^		*p*‐value	SEM
	CM	SM		
Eating time	208.51^a^	157.89^b^	0.03	13.19
Ruminating time	47.61^b^	76.07^a^	0.01	6.91
Nonchewing behavior	390.75	411.54	0.36	10.08
Drinking time	16.00	14.50	0.33	0.67

1 CM = control mix of 0.244 g/kg dry matter (DM) monensin + 0.195 g/kg DM virginiamycin; SM = standard mix of 5.848 g/kg DM Fator P^®^ + 0.122 g/kg DM monensin. ^a,b^Means followed by different letters differed by Tukey's test at 5% significance.

SEM, standard error of the mean.

The DMI differed among treatments (*p* < 0.05) in both the growing and finishing trials. In the growing trial, the DMI of animals fed with SM was higher than that of animals fed with CM. During the finishing trial, the same pattern was observed, with the DMI generally being lower in the animals fed with CM relative to the other treatments (SM, FPVM, and FP). The distribution observed in the total feedlot period was similar to that observed in the finishing trial (Table 3).

**Table 3:** Dry matter intake, average daily gain, feed conversion ratio, and final body weight during the growing and finishing trials, and in the total feedlot period, under different dietary treatments

Variable	Treatment^1^					
	CM	SM	FPVM	FP	*p*‐value	SEM
Dry matter intake (%BW)						
Growing	2.38^b^	2.52^a^	–	–	0.04	0.03
Finishing	2.03^b^	2.30^a^	2.39^a^	2.34^a^	0.004	0.05
Total	2.15^b^	2.40^a^	2.44^a^	2.37^a^	0.005	0.04
Average daily gain (kg/day)						
Growing	0.775	0.873	‐	‐	0.36	0.04
Finishing	1.177	1.298	1.219	1.343	0.28	0.03
Total	1.093^a^	1.193^a^	0.950^b^	1.180^a^	0.001	0.03
Feed conversion ratio						
Growing	12.77	12.35	–	–	0.80	0.67
Finishing	8.31	8.74	9.40	8.57	0.24	0.19
Total	9.48^b^	9.32^b^	11.50^a^	9.34^b^	0.002	0.28
Final body weight (kg)						
Growing	431.25	433.72	–	–	0.69	2.50
Finishing	541.38^b^	547.75^ab^	516.13^c^	548.25^a^	*	5.00

1 CM = control mix of 0.244 g/kg dry matter (DM) monensin + 0.195 g/kg DM virginiamycin; SM = standard mix of 5.848 g/kg DM Fator P^®^ + 0.122 g/kg DM monensin; FPVM = 5.848 g/kg DM Fator P^®^ + 0.195 g/kg DM virginiamycin; FP = 5.848 g/kg DM Fator P^®^. ^a,b,c^Means followed by different letters were different by Tukey's test at 5% significance. *p <* *0.0001.

SEM, standard error of the mean.

The animal performance as measured by ADG did not differ (*p* > 0.05) among the treatments during both the growing and finishing trials (Table 3). However, when the total feedlot period was considered, a significantly lower ADG was observed in the animals fed with FPVM (0.950 kg/day) than in those fed with the other treatments (1.093, 1.180, and 1.193 kg/day for CM, FP, and SM, respectively; *p* = 0.001). Likewise, the final body weight (FBW) did not differ between the CM and SM treatments (mean FBW of 432.49 kg; *p* > 0.05) in the growing trial. However, during the finishing trial, the animals fed with FP had the highest FBW (548.25 kg), followed by SM (547.75 kg, difference with FP not significant), CM (541.38 kg), and FPVM (516.13 kg; *p* < 0.0001).

Similar distributions were observed for the FCRs (Table 3), in that no differences (*p* > 0.05) were found between the growing and finishing trials. However, over the total feedlot period, the animals fed with FPVM had significantly higher FCR values (11.50; *p* < 0.05) than those in the other treatment groups (9.48, 9.34, and 9.32 for CM, FP, and SM, respectively).

With regard to the AD of nutrients, that of DM was similar (*p* > 0.05) at every trial (Table 4). When the AD of CP was evaluated in the growing trial and in the total feedlot period, no differences were observed among the treatments (*p* > 0.05). However, differences were observed in the finishing trial (*p* = 0.02), where the animals fed with FP showed a higher AD of CP than those fed with SM.

**Table 4:** Apparent digestibility (AD) of nutrients in animals during the growing and finishing trials, and in the total feedlot period, under different dietary treatments

Variable	Treatment^1^				*p*‐value	SEM
	CM	SM	FPVM	FP		
AD dry matter (%)						
Growing	49.16	46.89	–	–	0.72	2.59
Finishing	73.14	71.06	72.98	74.68	0.52	0.80
Total	64.59	64.20	62.59	64.09	0.93	1.03
AD crude protein (%)						
Growing	63.17	62.26	–	–	0.79	1.37
Finishing	73.90^ab^	66.23^b^	70.83^ab^	76.30^a^	0.02	1.36
Total	70.07	65.05	67.82	71.01	0.13	0.99
AD neutral detergent fiber (%)						
Growing	47.24^a^	35.19^b^	–	–	0.002	1.89
Finishing	70.66^a^	70.05^a^	72.31^a^	58.58^b^	0.001	1.88
Total	64.04^a^	55.41^ab^	54.26^ab^	45.24^b^	0.03	2.48

1 CM = control mix of 0.244 g/kg dry matter (DM) monensin + 0.195 g/kg DM virginiamycin; SM = standard mix of 5.848 g/kg DM Fator P^®^ + 0.122 g/kg DM monensin; FPVM = 5.848 g/kg DM Fator P^®^ + 0.195 g/kg DM virginiamycin; FP = 5.848 g/kg DM Fator P^®^. ^a,b^Means followed by different letters were different by Tukey's test at 5% significance.

SEM, standard error of the mean.

Differences in the AD of NDF were observed (*p* = 0.002) at each trial. During the growing trial, animals fed with CM (47.24%) had a higher AD of NDF than did those fed with SM (35.19%). For the finishing trial, there were also differences (*p* = 0.001) between animals fed with the different diets, where the value for FP was the lowest. When the total feedlot period was evaluated, the greatest differences in the AD of NDF (*p* = 0.03) were observed between the animals fed with CM (64.04%) and FP (45.24%), whereas similar values were found with SM and FPVM (Table 4).

With regard to the ruminal parameters, only the NH_3_‐N contents (*p* = 0.002) and acetic acid:propionic acid ratios (*p* = 0.009) showed significant differences among the dietary treatments (Table 5). However, trends were found for the pH (*p* = 0.07) and butyric acid concentrations (*p* = 0.05) in terms of the different treatments, and likewise for the pH (*p* < 0.0001) and propionic acid concentrations (P = 0.02) in terms of the different sampling times. The pH values were lower at 2 and 16 h after feeding with FPVM (6.25) and FP (6.19), respectively. The changes in propionic acid concentrations were significant for SM (4.04 mmol/L), CM (3.66 mmol/L), and FP (2.57 mmol/L), respectively, at 0 and 24 h (the time for the latter two treatments). Nonsignificant trends were observed for the NH_3_‐N, acetic and butyric acid, and total VFA concentrations (*p* = 0.08, *p* = 0.09, *p* = 0.07, and *p* = 0.05, respectively). No treatment × time interactions were observed for any of the variables (*p* > 0.05).

**Table 5:** Rumen parameters [pH, ammonia nitrogen (NH_3_‐N), and volatile fatty acids] of animals fed different experimental diets during the finishing trial of the feedlot

Variable	Treatment^a^				SEM	*p*‐value		
	CM	SM	FPVM	FP		Tr	Time	Tr × T^b^
pH	6.75	6.72	6.51	6.58	0.04	0.07	*	0.97
NH_3_‐N(mg/dL)	4.37	2.97	6.43	7.12	0.43	0.002	0.08	0.75
Volatile fatty acids (mmol/L)								
Acetic acid	22.44	25.67	29.32	29.20	1.33	0.21	0.09	0.45
Propionic acid	8.18	8.62	10.42	8.66	0.53	0.44	0.02	0.59
Butyric acid	3.32	5.04	4.33	5.08	0.26	0.05	0.07	0.38
Total	35.68	41.34	47.92	47.64	2.28	0.18	0.05	0.62
Acet:Prop^c^	2.94	3.13	2.82	3.51	0.07	0.009	0.24	0.95

a CM = control mix of 0.244 g/kg dry matter (DM) monensin + 0.195 g/kg DM virginiamycin; SM = standard mix of 5.848 g/kg DM Fator P^®^ + 0.122 g/kg DM monensin; FPVM = 5.848 g/kg DM Fator P^®^ + 0.195 g/kg DM virginiamycin; FP = 5.848 g/kg DM Fator P^®^.

b Effect of interaction between the treatment (Tr) and time (T).

c Acetic acid:propionic acid ratio.

*p <* *0.0001.

SEM, standard error of the mean.

## 4 Discussion

Evaluation of the animal feeding behaviors during the growing trial is important for determining how many cattle adapt to diets. If the animals have not had an adequate eating time, changes made to the diet management can give an ideal food intake return. The lower ruminating time (RT) observed for animals fed with CM may be due to an increased ruminal passage rate, as described by Bateman et al. (2004). Notably, the higher eating time of these CM‐fed animals did not result in a higher DMI; therefore, their lower feed intake per meal may be related to a higher ruminal passage rate. A similar feeding behavior was noted by Nagajara and Titgemeyer (2007), in their review of the study by Erickson et al. (2003), where monensin tended to reduce the intake rate and meal size while increasing the number of daily meals. The feeding behavior of animals fed with SM showed that the combination of Fator P^®^ with a traditional additive (monensin) can result in a higher RT owing to improvement in the rumen environment brought about by the lower O_2_ availability (Morais, Berchielli, & Reis, 2011), which favors ruminal digestion and animal performance.

The lower DMI observed in animals fed with CM is consistent with the main effect of monensin, an ionophore that reduces the DMI in diets with high concentrate levels (Castillo et al., 2004). The DMI was higher with the treatments containing Fator P^®^ during both trials and in the total feedlot period, which may be due to the reduced O_2_ level inside the rumen. According to Wallace (1994), and as noted by Morais et al. (2011), the yeast *S. cerevisiae* will consume O_2_ through respiration inside the rumen when supplied in ruminant diets. This process provides a better environment for anaerobic ruminal microorganisms, which could result in higher DMIs.

The ADG of animals fed with SM, CM, and FP, when evaluated over the total feedlot period, was higher than that observed for animals fed with FPVM (0.950 kg/day). The lower ADG with FPVM was probably caused by a negative interaction between the two main compounds in this mix (Fator P^®^ and virginiamycin). Because yeast is aerobic and cannot survive for long periods inside the anaerobic condition of the rumen environment, its continuous supply is needed to maintain a minimum effective concentration of 10^5^ colony‐forming units (Jouany & Morgavi, 2007). Such maintenance of these yeast cells inside the rumen is a challenge, and their gradual decrease and absence can cause low animal performance. Therefore, the use of a nonionophore supplement such as virginiamycin can challenge the maintenance of a high yeast level.

On the other hand, it should be highlighted that the other treatments containing Fator P^®^ (SM and FP) resulted in similar ADGs during the growing and finishing trials and in the total feedlot period, being higher than the ADG obtained with the traditional additive (CM). Several factors affected the results obtained with the use of a probiotic in the ruminant diets, such as the yeast strain and dose and the diet composition. However, several authors have reported that the use of probiotics (*S. cerevisiae*) did not change or improve the ADG (Fernandes, D’Aurea, Garcia, & Neto, 2015; Sartori et al., 2017; Vohra, Syal, & Madan, 2016).

The main effect of a traditional additive (monensin) is to improve the FCR (Nagajara & Lechtenberg, 2007; Vohra et al., 2016), which was indeed observed for the total feedlot period in our study. Animals fed with this compound and with the organic additive only (CM, SM, and FP) presented better FCR values (average ~9.30) than those of the animals fed with FPVM (FCR = 11.50). The poor results with FPVM are probably related to the negative effect caused by virginiamycin in relation to *S. cerevisiae*. Cocito (1979) reported that virginiamycin, a nonionophore additive, blocks protein synthesis by binding to the 50S subunit of the ribosome. When considering that Fator P^®^ contains a beneficial microorganism (*S. cerevisiae*) for improving the ruminal condition, diets combining these two additives would result in an overall negative effect compared with the effects of each individual additive alone. Thus, the simultaneous use of virginiamycin and Fator P^®^ does not result in adequate FCR values, in contrast to the other treatments (CM, SM, and FP).

Some authors have noted that the results obtained when yeasts are used in ruminant diets depend on many factors, including the yeast strain and dose, and diet composition (Newbold, Wallace, Chen, & McIntosh, 1995; Williams, Tait, Innes, & Newbold, 1991). In addition, blends that included Fator P^®^ affected the responses among different treatments. It is probable that the essential fatty acids in Fator P^®^ are responsible for the lower AD of NDF in each trial (growing, finishing, and total feedlot period). Several mechanisms could be involved in the action of fatty acids in rumen fermentation; however, antimicrobial effects and food particle coating (Nagaraja et al., 1997) may be important in explaining the results observed for the AD of NDF. Therefore, considering that the feedlot diets were composed essentially of concentrate, the effect on the AD of NDF was expected.

With regard to the AD of CP, the association between Fator P^®^ and monensin can result in decreased protozoa activity, mainly of *Entodinium* spp. and *Enoplopastron* spp. (Morais et al., 2011). Rumen protozoa possess protease (Forsberg, Lovelock, Krumholz, & Buchanan‐Smith, 1984), peptidase (Newbold, McKain, & Wallace, 1989), and deaminase (Wallace, McEwan, McIntosh, Teferedegne, & Newbold, 2002) activities; therefore, the AD of CP with the SM diet would be reduced as a result of less protein degradation (Faciola & Broderick, 2014), compared with that observed with the other treatment additives.

According to Santos (2011), ruminal acidosis is caused by abrupt changes in the diet and normally occurs in animals fed with diets containing a high concentrate level, as used to feedlots. In this system, the aminolytic bacteria are the main species present in the rumen environment. Santos (2011) reported that these species grew better in a pH interval that ranged from 5.5 to 6.5. Considering this information, all the additives studied could keep the pH at an adequate level, as none of the sampling times or treatments in this study reached pH values of below 6.19.

The values obtained for NH_3_‐N showed a similar trend to those for the AD of CP, strengthening the theory of decreased protozoa activity (Morais et al., 2011). The ruminal NH_3_‐N content in animals fed with SM was low, at 2.97 mg/dL. However, Satter and Slyter (1974), Schaefer, Davis, and Bryant (1980), and Pengpeng and Tan (2013) reported that the optimal concentration range of NH_3_‐N varies from 2.5 to 18 mg/dL to satisfy microbial growth requirements. The NH_3_‐N value with SM observed in our study was 4.16 mg/dL. Therefore, the use of Fator P^®^ in combination with monensin could result in higher bypass protein for the lower digestive tract, improving the protein availability for the host. This could provide better conditions for animal performance, as observed for the ADG (1.193 kg/day) observed in this study.

Studies have reported varied effects of yeasts on VFAs (Vohra et al., 2016). The reasons for this could be due to yeast‐related factors (e.g., the amount of yeast culture fed, and the strain) or animal‐related factors (e.g., the age and physiological status of animals fed on a yeast‐supplemented diet). The low propionic acid concentration may be due to the composition of Fator P^®^, and more specifically its essential fatty acids (linoleic and oleic acids). Similar results were reported by Evans and Martin (2000), who evaluated thymol, which is an essential oil rather than a fatty acid. The authors reported depletion of the acetic acid and propionic acid concentrations in their in vitro study. In our present study, it is probable that the period between meals led to the reduced propionic acid concentration at specific sampling times (0 and 24 h after feeding).

The reduced propionic acid concentration may have affected the acetic acid:propionic acid ratio. As observed by Abd El‐Tawab, Youssef, Bakr, Fthenakis, and Giadinis (2016), the effects of probiotics on VFAs are not fully understood. The acetic acid:propionic acid ratio is better when close to 1.00 (Berchielli, Pires, & Oliveira, 2011), owing to the energetic losses associated with methane gas being lower, and additional energy is therefore available for animal performance.

Taken together, our results indicate that the organic additive Fator P^®^ can be used during the growing trial to improve the DMI and rumination time. Considering the prohibition of traditional additives in animal feed by various countries, Fator P^®^ would be a viable and safe strategy for supplementation to beef cattle finished with high‐concentrate diets in feedlot systems.
